# Working alliance and its relationship to outcomes in a randomized controlled trial (RCT) of antipsychotic medication

**DOI:** 10.1186/1471-244X-13-28

**Published:** 2013-01-15

**Authors:** Til Wykes, Diana Rose, Paul Williams, Anthony S David

**Affiliations:** 1 NIHR MHRN and Institute of Psychiatry, King's College London, De Crespigny Park, London SE5 8AF, UK; 2 Service User Research Enterprise, Institute of Psychiatry, King's College London, De Crespigny Park, London SE5 8AF, UK; 3 Institute of Psychiatry, King's College London, De Crespigny Park, London SE5 8AF, UK; 4 Department of Psychology, Institute of Psychiatry, King's College London, De Crespigny Park, London SE5 8AF, UK

**Keywords:** Injectable medication, Patient empowerment, Therapeutic alliance, Clinical relationship, Schizophrenia

## Abstract

**Background:**

Long acting injections (LAI) have been associated with perceptions of coercion in cross sectional studies but there have been no longitudinal studies of the effects on clinical relationships with newer depot medications.

**Method:**

Randomized controlled trial with (50) participants with a diagnosis of schizophrenia randomized to risperidone LAI or oral atypical antipsychotic medication. The main outcome was the Working Alliance Inventory (WAI) with background variables (symptoms, side effect, social functioning, quality of life) measured before randomization and at two years.

**Results:**

At follow-up (14 risperidone LAI and 16 oral medication) analyses including predictors of missing data and baseline score showed a trend for those on risperidone LAI to reduce WAI score and those on oral medication showing no change. Sensitivity analyses showed (i) a significant detrimental effect of LAI on WAI and (ii) the pattern of results was not affected by change in symptoms over the study.

**Conclusion:**

This is the first study to show that the prescription of depot atypical depot medication is associated with detrimental effects on clinical relationships after 2 years of continual treatment.

## Background

Working alliance is the name given to the therapeutic relationship between a clinician and a patient. A good therapeutic relationship between clinician and patient is purported to be vital for a good outcome [1] and across all psychiatric disorders there is good supporting evidence that working alliance is a significant predictor of broad based outcomes which is not moderated by the severity of the psychiatric disorder [2,3]. Working alliance refers to the relationship between the clinician and the patient on such issues as goals and means of achieving goals.

In people with a diagnosis of schizophrenia, therapeutic alliance predicts successful outcome from cognitive behavioural therapy [4,5]. Poorer working alliance has been found to be related to increased symptom severity and, in some studies, reduced social ability [6-9].

Evidence for long acting injectable (LAI) or depot medication affecting the therapeutic relationships is mixed. Some patients perceive long-acting depot antipsychotics to be coercive [10,11] while many clinicians maintain that depots are underutilized as they offer advantages in terms of convenience, enhanced relapse prevention with reduced rehospitalization rates [12-15]. Intriguingly in a recent meta-analysis of oral versus depot medication studies, Leucht and colleagues showed that relapse was reduced for people on depot medication although oral medication non-adherence was rated as no greater than with depot. It may be that clinicians are over-estimating adherence in those on oral medication compared to depot where compliance is easily monitored.

The ability to monitor medication compliance may fuel patients' negative attitudes to medication. For instance, Patel and colleagues [16] found that patients’ negative attitudes to depot compared to oral medication, including the experience of coercion [17], was related to perceptions of being forced to take medication. These authors suggest that perceived coercion might be alleviated if the decision on prescribing is discussed using non-threatening language and with respect given to patient’s illness beliefs. Proper attention should also be given to patient choice. However, it should be noted that the patients in that and prior studies would have had all or most of their experience with first generation ‘typical’ depots which are likely to have more extra-pyramidal side effects than second generation or ‘atypical’ antipsychotics.

The study here investigates whether being placed on long acting atypical antipsychotic medication leads to worsening of the therapeutic alliance, as measured using the Working Alliance Inventory (WAI), in relation to oral atypical antipsychotic medication. This was a multi-centre, multi country, industry-sponsored clinical trial. The UK arm was hosted in the National Health Service (NHS) by infrastructure called the National Institute for Health Research (NIHR) Mental Health Research Network (MHRN) [18]. The industry partner agreed to include a service user valued outcome in addition to the main (illness exacerbation) and secondary outcomes in their protocol, namely a measure of therapeutic alliance. The aim of this add-on study was to investigate whether effects on the relationship between clinician and patient depended on the trial arm.

In this RCT, the participants knew that they were entering a study where the treatment decision would be made by the randomization programme. While they must have accepted in principle that they may be placed in the long acting injection arm, as part of the informed consent process of the trial, the actual decision to do so would have been made independently. Thus the effect of having a LAI on the therapeutic alliance can be studied prospectively in relative isolation of other clinical factors. The study benefits significantly from a randomized design which controls for any factors that would normally be associated with clinicians and patients reaching a consensus about commencing depot treatment. The current study also has the advantage of comparing LAI and oral atypical agents thus matching broadly for the range of likely side effects. Finally, because severity of distress influences ratings on the WAI we also report associations between the scale and a range of standard measures of symptoms, illness severity and level of functioning fundamental to psychotic disorders.

### Aims

To assess whether the experience of atypical LAI is associated with changes in clinical alliance.

## Method

### Design

Data for this paper were drawn from a randomized, open-label, parallel-group, multi-country and multi-centre study of risperidone LAI versus oral atypical antipsychotics (AAP). Only data from the UK are included here as this centre only collected measures of therapeutic alliance. Assessments used in these analyses are taken from the baseline and final outcomes which is when the therapeutic alliance was measured. In between these assessments there were four other assessment points when clinical exacerbations were measured. The design was posted on ClinicalTrials.gov, NCT00256997; sponsor Janssen-Ortho Inc., Canada.

#### Ethics

The study complied with the Helsinki Declaration ( http://www.wma.net/en/30publications/10policies/b3/index.html). Research Ethics Committees of Northern Ireland (ORECNI) Ref number 05/NIR05/41.

### Participants

Detailed entry criteria are shown on the registered trial protocol. The ones of relevance in this study are:

· DSM-IV TR diagnosis of schizophrenia.

· currently treated with oral AAP medication.

· aged 18 – 65 years.

· have had at least 2 hospitalisations or clinical exacerbations over the past 2 years due to suspected poor adherence.

· non-treatment resistance shown by a satisfactory response to oral antipsychotics.

All participants gave written informed consent.

### Measures and procedure

All measures were collected independently of clinical teams by trained researchers who interviewed participants or who collated the self report data.

(i) *Main outcome of interest* - *Therapeutic Alliance*: *Working Alliance Inventory*:

Working Alliance Inventory (WAI [19]); is a 36-item self report measure completed by all participants about their relationship with their prescribing psychiatrist. Each item is measured on a seven point scale of −3 to +3. Higher scores indicate better working alliances. Cronbach’s alpha for this client completed scale is reported as 0.93 and for items in the current smaller sample was 0.79.

(ii) *Symptoms*:

Positive and Negative Symptom Scale (PANSS; [20]) divided into Positive, Negative, General and Global symptoms; Clinical Global Impression Severity (CGI-S; [21]) which is a 7 point scale (1 = mild symptom levels, 7 extremely severe) and Clinical Global Impression Change (CGI-C) which is also a 7 point scale (1 = much improved, 7 = very much worse).

(iii) *Quality of life*:

Assessment of Quality of Life (AQoL; [22]) measures five dimensions; illness, independent living, social relationships, physical senses and psychological wellbeing. Cronbach’s alpha 0.81 for the whole scale.

(iv) *Functioning*:

Personal and Social Performance Scale (PSP; [23]) The PSP is a 100-point single-item rating scale, subdivided into 10 equal intervals. The scale covers four main areas: socially useful activities, personal and social relationships, self-care and disturbing and aggressive behaviours.

(vi) *Clinical Exacerbations*:

These are defined as one of the following:

· Hospitalization due to an exacerbation of participant's schizophrenia.

· A change from current antipsychotic or initiation of an adjunctive antipsychotic treatment.

· a 2-point worsening in CGI-S plus at least one of the following (emergency or exceptional treatment as a result of clinical exacerbation, increased dose of medication as a result of poor symptom control, deliberate self-injury or emergence of clinically significant suicidal or homicidal ideation, violent behaviour resulting in injury or property damage).

### Statistical analyses

(i) ***Missing data***: If scale items for WAI were not complete then we prorated the scores as long as more than 80% of items had been completed. The assumption that participants were missing at random was investigated using univariate logistic regression analyses using demographic predictors which were then included in the longitudinal analyses otherwise we assumed that a complete case analysis is appropriate. For some analyses missing data at baseline was replaced by total sample mean and at follow-up the mean overall baseline WAI scores pre-randomization was used in the imputation. Methods of imputation were investigated in sensitivity analyses.

(ii) **Baseline relationships** between symptoms, quality of life and social functioning were explored using correlations.

(iii) **Changes in WAI** over time were investigated using paired t-tests and the effect of trial arm on WAI change using ANCOVA with baseline WAI as one covariate. As the relationship between clinician and patient is likely to be affected by clinical change over time we included whether a clinical exacerbation had occurred after 12 weeks (i.e. after a stable dose of medication had been achieved) as a further covariate in all analyses. Any predictor of missingness was also included in the model.

All analyses were carried out using STATA 11.2 (StataCorp).

## Results

54 participants were recruited to the study but 4 dropped out or were withdrawn prior to the baseline assessment and prior to randomization leaving 50 people who completed WAI at baseline. After pro-rating, 2 participants were not scored because they failed to complete >80% of the items, leaving 48 participants. At follow-up 30 people completed enough of the WAI items (i.e. above 29) to achieve a WAI score (14 risperidone LAI and 16 oral medication group). The socio-demographic and clinical characteristics of the participants at baseline are shown in Table 1. The groups, as expected from randomization, were relatively well balanced although the depot group were slightly younger than the oral medication group (36.9 vs 42.6) but this was not a significant difference (Table 1).

**Table 1:** Sample characteristics, main outcome and background variables; Average, [proportion] standard deviation

	**Risperidone LAI Group n** = **26**		**Oral Medication Group n** = **24**		**Total group n** = **50**
**Demographic variables**					
Age, years	36.8 (10.81)		42.58 (10.71)		39.6 (11.04)
Gender	73% Male,		70% Male		72% Male,
Ethnicity	77% White,		54% White,		66% White,
Substance use/abuse	46% No,		58% No		52% No,
**Clinical symptoms**					
	**Median** (**IQR**)		**Median** (**IQR**)		**Total Group**
	**Baseline**	**Follow**-**up**	**Baseline**	**Follow**-**up**	**Baseline**
PANSS total	80 (67–91)	71.5 (51–88)	71 (62.5-86)	60 (41–86)	80 (65–90)
PANSS Positive	16.5 (14–26)	15.5 (12–22)	17 (13–21)	15 (10–21)	18 (13–22)
PANSS Negative	21 (14–25)	17 (13–21)	20 (17.5-25)	16 (11–22)	21.5 (17–27)
CGI-Severity	4.5 (4–5)	3.5 (3–5)	4 (3–5)	3 (2–4)	4 (3–5)
AIMS	0 (0–1)	0 (0–0)	1 (0–3.5)	0 (0–1)	0 (0–3)
**Quality of life**					
AQOL Illness	0.42 (0.17-0.52)	0.52 (0.42-0.64)	0.36 (0.14-0.59)	0.42 (0.26-0.53)	0.40 (0.15-0.52)
AQOL Activities Daily Life	0.78 (0.62-0.0.86)	0.88 (0.78-1.00)	1.00 (0.78-1.00)	1.00 (0.79-1.00)	0.79 (0.62-1.00)
AQOL Social	0.70 (0.27-0.82)	0.74 (0.35-0.88)	0.69 (0.24-0.88)	0.74 (0.35-0.94)	0.69 (0.24-0.83)
AQOL Physical	1.00 (0.82-1.00)	0.93 (0.82-1.00)	1.00 (0.94-1.00)	1.00 (0.94-1.00)	1.00 (0.88-1.00)
AQOL Psychological	0.87 (0.73-0.91)	0.91 (0.73-0.93)	0.87 (0.67-0.93)	0.95 (0.83-1.00)	0.87 (0.70-0.93)
	Mean (sd)	Mean (sd)	Mean (sd)	Mean (sd)	Mean (sd)
**Working Alliance** (**prorated**)	48.33 (33.10)	27.30 (27.90)	45.97 (31.64)	49.30 (35.32)	48.20 (32.08)

The participants were mostly men, of white origin who were middle aged which is representative of those who have had some long term treatment which has not been particularly effective. They also had moderately severe symptoms and marked social and personal difficulties which were correlated (r = −0.56, p < 0.001).

Despite the possible barriers to a good therapeutic alliance (poor social functioning and higher levels of symptoms) the majority of WAI scores were positive which indicates a good working relationship. Only two individuals at baseline indicated that it was poor. Given that this group of participants would have been selected by their clinical teams as being potentially interested in taking part in a trial, it is likely that part of the judgement of potential participation was based on the quality of the therapeutic relationship.

### Baseline assessments

#### Identification of predictors of missingness

Potential specific predictors (Trial arm and baseline WAI) were explored and neither showed a significant effect. At follow-up 11 participants from risperidone LAI and 7 from the oral trial arm were missing (χ^2^ (1) = 0.94, p = 0.33) and baseline WAI was explored in a regression analysis but was not a significant predictor (p = 0.26). Exacerbations at 12 weeks which was intended as a covariate was a significant predictor of missing outcome measure at follow up (χ^2^ (1) = 4.16, p = 0.041) and was retained as a covariate. No other patient measures (including demographic (age, gender, ethnicity) clinical status (PANSS, CGI, AIMS, PSP) or Quality of Life (AQOL subscales)) were significantly associated with missing data at follow up.

#### Are there relationships between clinical variables and clinical alliance?

No demographic variables were related to baseline WAI scores (n = 48). Given previous research it might be expected that poorer social functioning and worse symptoms would be related to working alliance but in these analyses there was only a significant correlation with total symptoms (total PANSS score r = −0.29 p = 0.046, n = 48); more symptoms, poorer working alliance. However, people with substance misuse had similar WAI scores to those who had no current substance misuse. In terms of aspects of quality of life, the only significant correlation with baseline WAI score was level of perceived physical problems (correlation r = 0.36, p = 0.013).

#### Changes over time

The presence of clinical exacerbations 12 weeks from treatment onset did not differ between those who completed therapy and completed the WAI (Risperidone LAI = 4, oral AAP = 3; Fisher’s Exact test p = 0.6).

The first longitudinal analysis showed no significant change of WAI over time (N = 30, baseline WAI = 43.2 (sd31), follow-up WAI 39.0 (sd 33) paired t = 0.73, df 29, p = 0.47). This result is unlike that for most other clinical variables which did improve over time either significantly or at a trend level. All were tested using Wilcoxon sign-rank test due to non-normality: Symptoms (N = 42, z = 2.34, p = 0.019) CGI (N = 43, z = 3.03, p = 0.003), side effects (AIMS N = 40 z = 2.49, p = 0.013); Quality of Life illness (N = 36, z = −2.12, p = 0.034) and Quality of Life activities of daily living (N = 36, z = −2.82, p = 0.005). The only variable not showing this pattern was social and personal functioning which showed no change (PSP N = 43 z = −1.08, p = 0.279). There were no relationships between symptom changes and WAI and no effect of trial arm on these clinical and quality of life measures.

**Figure 1:**
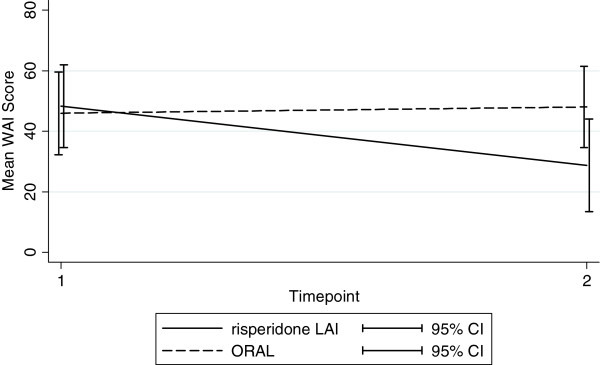
**Predicted mean WAI scores at time 2 from the regression model after adjusting for trial arm**, **exacerbations at 12 weeks and baseline WAI scores.**

The second set of analyses concentrated on testing whether the treatment arm affected follow-up scores on WAI. The analyses of WAI change over time included only complete cases and after prorating of scores there were 30 participants remaining (risperidone LAI 14, oral medication 16). In the ANCOVA WAI score at follow-up was the dependent variable, treatment group was the independent variable and baseline WAI and presence of an exacerbation were covariates in a linear regression model with robust standard errors. The results show a trend for a treatment group effect (Treatment group coefficient = 19.29, p = 0.076, 95% CI −2.20 to 40.78). The effect is illustrated in Figure 1. For the risperidone LAI group working alliance reduces over the trial whereas the oral group does not change. In the model baseline WAI was significant (coefficient = 0.55, p = 0.006, 95% CI 0.18 to 0.94) but exacerbations after 12 weeks was not.

We also carried out a sensitivity analysis to include all participants under the assumption that missing follow up data would be the average baseline WAI score. This sample contained 2 participants whose baseline scores were missing (one per trial arm) so these were also imputed as the baseline WAI score. In this model (N = 50) the effect of trial arm was significant (coefficient 13.66, p = 0.048, 95% CI 0.13 to 27.19) with WAI falling in the LAI group. Again baseline WAI was significant (coefficient = 0.34, p = 0.006, 95% CI 0.10 to 0.58) and exacerbations at 12 weeks and the missing follow up indicator were not.

A further sensitivity analysis that adjusted for change in PANSS score from baseline to follow up was performed on the model conducted for the primary analysis i.e. regression of WAI score at follow up on trial arm, exacerbations at 12 weeks, baseline WAI score and PANSS change from baseline as covariates (N = 30). This further analysis was to adjust for any change in symptoms throughout the study that may have an effect on the working alliance. When this was entered into the model the same pattern of results remained - trend for an effect of treatment on change in WAI (coefficient = 19.58, p = 0.074, 95% CI −2.01 to 41.18). In this model baseline WAI was significant (coefficient = −0.19, p = 0.007, 95% CI 0.16-0.23) but exacerbations after 12 weeks and PANSS change from baseline were not.

## Discussion

The study was unusual in that participants were taking part in a trial that would test the effectiveness of different drug formulations over a two year period. Most studies only follow participants up for relatively short periods of time and so do not uncover possible changes in service user valued outcomes. It is assumed that working alliance develops over a period of time and as such any changes will only be detected over periods of longer than one year. This trial offered an opportunity to investigate such changes. The participants entering the study had been in touch with their clinical teams for some time and already had a reasonably good therapeutic relationship. It is likely that it was this good relationship that led individuals to feel they would consent to the study. But it also suggests that it might be difficult to lift the level further.

In our study therapeutic relationship at baseline was related to symptom severity which has been reported previously [6-9]. This same pattern of relationships was also found at the end of the trial suggesting it is robust to changes in symptom level (r = −.443, df 1, p = 0.016). But unlike other studies we did not find a relationship with social functioning although previous evidence of such a relationship has not been consistent. This might be because of differences in measurement or the fact that social functioning in this group has little variation making subtle effects difficult to identify.

### Is there an effect of trial arm on therapeutic alliance?

All the clinical indices measured in this study improved over the course of the study but the trial arms could not be distinguished on these measures.

Working Alliance did show a trend for a treatment effect in the complete case analysis and this trend became significant in the imputations model. This change was a decrease in WAI only in the risperidone LAI arm of the trial. There were no changes in the oral arm. This is despite changes over time favouring improvements in the two groups.

Poorer working alliance being related to receiving depot medication is assumed because most data on depot medication show higher feelings of coercion which is not consistent with a good therapeutic relationship [10,11]. However, all this evidence comes from cross sectional studies investigating groups who have often received depot medication for some time. The difference in this study is that for the first time working alliance was measured directly and not inferred from coercion data. In addition, this is the first time that an investigation was carried out as individuals change from one oral medication to a depot. The fact that patients receiving depot medication perceive a loss of therapeutic relationship is certainly important clinically. It may be that depot medication has positive outcomes clinically, although in our study we could not distinguish the groups on symptoms or side effects, but decreases in clinical alliance might counteract these clinical gains in terms of translating them into real life functioning outcomes. Unfortunately perceived coercion was not measured in this study so we cannot say that this mediates (or is mediated by) a change in working alliance.

A good working alliance can be regarded as positive in and of itself as well as being a vehicle for aiding service users to follow treatment protocols (see [24]). In our study the trend for a reduction in working alliance associated with depot medication would not have had the latter effect since missing an injection would have been interpreted as a study violation and essentially drop-out. However, it may lead to reductions in compliance with other helpful treatment protocols such as recovery programmes or psychological treatments. This is a topic for future research.

### Strengths and limitations

A strength of the study is its longitudinal design and randomized treatment arms which provide methodological rigour. Previous studies of patients attitudes towards depots have often been cross sectional and because of a lack of randomization are limited by medication decisions being based on medical need. We also had a long period over which to assess an outcome which is likely to take months to develop and change. However, many patients considered for LAI will not have been able or willing to participate in an RCT design, because of comorbidities, instability or perhaps a very poor therapeutic alliance with their clinical teams, hence the generalizability of the study may be limited. The sample was mainly male and Caucasian which limits generalizability. There is a loss of statistical power through missing follow-up data resulting from such long follow-up. So in order to be rigorous we not only investigated missingness thoroughly but we also tested our assumptions, included any predictors of missingness and carried out sensitivity analyses. Our imputation model did show a significant effect and this result fits with other data on feelings of coercion experienced by people with psychosis who are prescribed depot medications. The worldwide study was terminated early because of very slow recruitment in some sites but we do not feel this was a limitation for this analysis as recruitment rates were reasonable in the UK. Clearly had we been able to continue recruitment a larger sample would have allowed more subtle effects to be detected and, of course, provide more substantive support for our finding. Finally the study did not record details of the therapeutic relationship and factors such as the number of face-to-face contacts and duration. Such factors may be important mediators of the WAI in this patient group.

We would not have been able to carry out this study without the vehicle of a randomized control trial sponsored by industry. The model of using current large scale Phase 3 or 4 studies to provide outcomes of importance to industry in addition to those of more academic or clinical interest provides for a clear future research partnership. In this study the company invested in an add-on study of specific interest to service users from the public and patient involvement arm of NIHR MHRN. This meant that the study was more appealing to both clinicians and service user alike and gave it even more clinical relevance. The NHS infrastructure provided by NIHR foster these partnerships with their high quality delivery mechanisms and well formed service user involvement.

## Conclusions

The study reports that there is a reduction in working alliance following the introduction of an LAI compared to continuing on oral medication. This is despite the clinical benefits achieved by both groups of patients. There are other important mediators and moderators that were not investigated but could provide clear advice on how to avert such effects.

### Clinical implications

The results of this longitudinal study suggest that possible deterioration of working alliance needs further attention when a patient has been prescribed a depot even when they participated in the decision making (i.e. agreement to participate in the trial). Mental health staff should also ensure that prescription of a depot is a true choice for the service user. The study design allows us to conclude that the prescription of an LAI is most likely to have produced a deterioration of working alliance and this is a strong argument for service user choice. Therapeutic relationships may also vary with the modality of medication in the sense that more attention is given by clinicians to those on oral medication to ensure adherence. For those on depot the contact may be limited to visits to specialised clinics where less time is spent on continued development of the therapeutic relationship. Future studies need to concentrate on whether improving the therapeutic relationship can of itself alleviate the negative effects of receiving LAIs found in this investigation.

## Competing interests

The data were independently analysed by the principal authors who report no conflicts of interest. The views expressed are those of the authors and not necessarily those of the NHS, the NIHR, Department of Health or Janssen Pharmaceuticals.

## Authors’ contributions

AD, DR and TW were involved in the initial concept and design of the study, PW led on the analysis approach and all authors carried out interpretation of the results and production of the paper and critically revised and agreed it before publication. All authors have read and approved the final manuscript.

## Pre-publication history

The pre-publication history for this paper can be accessed here:

http://www.biomedcentral.com/1471-244X/13/28/prepub
